# Genetic Traits of *Vibrio cholerae* O1 Haitian Isolates That Are Absent in Contemporary Strains from Kolkata, India

**DOI:** 10.1371/journal.pone.0112973

**Published:** 2014-11-21

**Authors:** Priyanka Ghosh, Arindam Naha, G. P. Pazhani, T. Ramamurthy, Asish K. Mukhopadhyay

**Affiliations:** Division of Bacteriology, National Institute of Cholera, Enteric Diseases, Kolkata, India; Universiteit Utrecht, Netherlands

## Abstract

The world's worst cholera epidemic in Haiti (2010) coerced to trace the origin and dissemination of the causative agent *Vibrio cholerae* O1 for proper management of cholera. Sequence analysis of the Haitian strain showed several variations in the genes encoding cholera toxin B subunit (*ctxB*); toxin-co-regulated pilus (*tcpA*), repeat in toxins (*rtxA*), quinolone resistance-determining region (QRDR) of gyrase A (*gyrA*), *rstB* of RS element along with the change in the number of repeat sequences at the promoter region of *ctxAB*. Our earlier studies showed that variant *tcpA* (*tcpA CIRS*) and *ctxB* (*ctxB7*) first appeared in Kolkata during 2003 and 2006, respectively. The present study revealed that a variant *rtxA* was first isolated in Kolkata during 2004 and probably formed the genetic background for the emergence of the *ctxB*7 allele as we were unable to detect a single strain with the combination of El Tor *rtxA* and *ctxB*7. The variant *gyrA* was first time detected in Kolkata during 1994. The Kolkata strains contained four heptad repeats (TTTTGAT) in their CT promoter regions whereas Haitian strains carried 5 heptad repeats. Haitian strains had 3 nucleotide deletions at the *rstB* gene, which is a unique feature of the classical biotype strains. But the Kolkata strains did not have such deletion mutations in the *rstB*. Our study demonstrated the existence of some Haitian genetic traits in Kolkata isolates along with the dissimilarities in genomic content with respect to *rstB* and *ctxAB* promoter region. Finally, we conclude that Haitian variant strain may be evolved due to sequential event in the Indian subcontinent strain with some cryptic modification in the genome.

## Introduction

Cholera is still a considerable health burden due to poor hygiene and sanitation in developing countries, especially in Africa and Asia. This severe, dehydrating diarrheal disease is triggered by the Gram negative bacterium *Vibrio cholerae*. Till date, *V. cholerae* has more than 200 established serogroups, but only O1 and O139 serogroups are responsible for epidemic and pandemic cholera [1],[2]. The O1 serogroup is further classified into two biotypes, namely, classical and El Tor. Since 1817, seven cholera pandemics have occurred in the recorded history. The classical biotype strains had instigated the first six pandemics, whereas the ongoing seventh pandemic has been caused by the El Tor biotype [2]. In recent years, novel pathogenic variants of *V. cholerae* O1 have been emerged and disseminated throughout the world [3]–[7]. This indicates a cryptic change in the genome of *V. cholerae* subsequently modified the epidemiology of cholera. The devastating cholera outbreak during 2010 in Haiti, for the first time in almost a century, placed this ancient scourge at the forefront of the global public health agenda [8]. In this outbreak, more than 500,000 were infected and around 8000 people died. Many published reports suggest that the origin of cholera in this region may be from Asian countries and/or due to indigenous strains [9]–[11]. The World Health Organization, in May 2011, documented the re-emergence of cholera as a substantial global public health problem and asked for the execution of an integrated and inclusive global approach to control the cholera [12].

Whole genome sequencing analysis of *V. cholerae* strain in Haiti revealed some mutations in different segments of their chromosomes [10]. These include structural variation in superintegron, VSP-2, and SXT as well as SNPs in the *ctxB* allele which codes for the B subunit of the cholera enterotoxin (CT) and also in the *tcpA* allele, which codes for the major structural protein of the toxin-co regulated pilus, the second major virulence factor of *V. cholerae* [13],[14]. Further analysis of the sequencing data and BLAST results showed presence of mutation in the *rtxA* gene encoding the multifunctional auto processing RTX toxin [15], quinolone resistance-determining region (QRDR) of gyrase A (*gyrA*), *rstB* encoding for protein required for phase DNA integration and part of CTX*Φ* [16] and change in the number of heptad repeats (TTTTGAT) at the promoter region of *ctx* [14]. Many published reports demonstrated that not only in Haiti, this variant type of *ctxB* (*ctxB7*) and *tcpA* (*tcpA CIRS*) allele were predominated throughout the world but they were highlighted after the disastrous Haitian cholera outbreak. Presence of *ctxB7* and *tcpA* CIRS allele was reported from the Nigeria cholera outbreaks in 2009 and 2010 [17]. Presence of *gyrA* ^Ser83→Ile^ was also presented in Nigerian outbreaks [17]. *ctxB7* allele was also the genomic backbone of the strains isolated from the cholera outbreaks in South-western India in 2012 [18]. Cholera outbreak in Mexico during 2013 also presented the *ctxB7* genotype [19]. Appearance and gradual dissemination of variant *ctxB* allele (*ctxB7*) in Kolkata from the year 2006 onward was presented in our previous study [4]. Our earlier studies revealed that the El Tor variant strains of *V. cholerae* O1 secreting classical CT have completely replaced the El Tor CT producing strains in Kolkata, India since 1995 [3]. El Tor type *ctxB* was also replaced by the classical allele in Bangladesh since 2001 was documented [20]. Presence of Classical CT producing El Tor strains in the US Gulf Coast was reported by Olsvik *et al* (1993) [21]. New *ctxB* genotypes were also observed in Zambia [22]. Our recent study depicted that the variant *tcpA* allele was first identified in Kolkata during 2003 and interestingly soon after its appearance, the new variant *tcpA* totally replaced the El Tor *tcpA* [23]. We further investigated the novel mutations, if any, in the *rtxA*, *gyrA*, *rstB* along with the change in the repeat number at promoter region of CT. In order to accommodate more number of strains for screening, we have developed a simple PCR-based assay utilizing allele specific primers to accurately discriminate the pure El Tor and variant type *rtxA*, *gyrA*, *rstB* alleles. PCR based results were further validated with the sequencing study using a collection of strains covering different years. In addition, retrospective analysis was performed with a large collection of strains from Kolkata. Our results highlight the existence of some genetic traits in Haitian variant *V. cholerae* O1 strains that are absent in contemporary strains from Kolkata.

## Materials and Methods

### Bacteriology and serology

A total of 237 representative *V. cholerae* O1 strains isolated between 2001 and 2012 were included for the analysis of *rtxA*, *rstB*, and *gyrA* genes. Additionally another 50 strains isolated between 1989 and 2000 were also included in the genotyping of *gyrA*. All the *V. cholerae* strains used for this study were selected from the strain repository of National Institute of Cholera and Enteric Diseases (NICED), Kolkata, India and were isolated from the hospitalized cholera patients. NICED Human Ethical committee had approved the study (Approval No. C-48/2012-T&E). All the participants provided their written consent to participate in this study and these documents were maintained in the Clinical Division of NICED. The content and consent procedure was also approved by the above committee. The strains were grown in Luria Bertani broth (Becton Dickinson, Sparks, MD, USA) for 18 hrs. and then streaked on Luria agar (Becton Dickinson, Sparks) plates. Identity of these strains was reconfirmed serologically by the slide agglutination with O1 specific polyclonal antiserum and serotype specific antisera (Becton Dickinson, Sparks). *V. cholerae* O1 strains EL-1786 (Ogawa), N16961 (Inaba) and 0395 (Ogawa) were used as standard strains for the Haitian, El Tor and classical type, respectively.

### Preparation of DNA template for PCR

One loopful of an overnight culture from LA plate was suspended in 200 µl of Tris-EDTA buffer (pH 8.0) and then lysed by vigorous vortexing with mixture of phenol: chloroform: isoamyl alcohol (25∶24∶1) saturated with 10 mM Tris and 1 mM EDTA. (Sigma-Aldrich, St Louis, MO, USA) Supernatant was collected carefully following centrifugation at 12,000 rpm for 15 min and was extracted once with 100 µl of mixture of chloroform: isoamyl alcohol (24∶1) and centrifuged for 15 min at 12,000 rpm. The supernatant containing the DNA was used as template for PCR analysis

### Development of new PCR assay

By exploiting the single base mutation, three new PCR assays were designed and validated in this study. We designed allele-specific either forward or reverse primer containing mismatch at the 3′ ends (Table 1). Three separate primers, which include one forward primer common for both El Tor and variant type *rtxA* alleles (*rtxA*-F) and two reverse primers (*rtxA*-R1 and *rtxA*-R2) specific for El Tor and variant type *rtxA* alleles, respectively were designed (Table 1). These allele-specific primers each carry specific nucleotide, C and T, for El Tor and variant type, respectively, at the 3′ end. Furthermore, we enhanced the 3′ mismatch effect by introducing another nucleotide alternation C (rather than A) at the second nucleotide from the 3′end of both the primers (Table 1). We also designed one forward primer common for both El Tor and variant type *gyrA* alleles (*gyrA*-F) and two reverse primers (*gyrA*-R1 and *gyrA*-R2) specific for variant and El Tor type *gyrA* alleles (Table 1). Primer for *rstB* allele was constructed.

**Table 1:** Primer sequences, amplicon size and annealing temperature used in PCR assays.

Primer	Sequence (5′-3′)	Amplicon (bp)	Annealing (°C)	Reference
rtxAR1	tgtgaaccacgtctgCC	187	54	This study
rtxAR2	tgtgaaccacgtctgCT	187	54	This study
rtxAF	atcggaatgagtgagaaagacc			This study
rtxAF'	tactttaatggtaaccgcgct	422	54	This study
rtxAR	cattgtcactgtacttacgtc			This study
gyrAR1	gatggtgtcgtaaaccgcTA	177	60	This study
gyrAR2	gatggtgtcgtaaaccgcTC	177	61	This study
gyrAF	tgctcttcctgatgtgcgtgatg	411	61.8	This study
gyrAR	ttgatcagcaggttcgggatc			This study
rstBF1	attctgaaggggtgagtCgta	160	58	This study
rstBR2	ctggtcatcgcgtcactggat			This study
cepF	gccaatcacggtaacaatca	820	48	This study
rstAR	aggaaattcacgacgattcac			This study
ZotF(S)	cgagctaccgctacaaggtgcta	470	55	Naha A *et al*. 2013
ctxAR(S)	cgtgcctaacaaatcccgtctgag			

### Sequencing Analysis

PCR for the respective segment from 16 *V. cholerae* O1 isolates was performed to determine the nucleotide sequence of the *rtxA*, *gyrA*, *rstB* and *ctxAB* promoter sequence with different set of primers (Table 1). Amplified products were purified using the Qiaquick PCR purification kit (QIAGEN, Hilden, Germany) and both the strands were sequenced in an automated sequencer. (ABI PRISM 3100 Genetic Analyzer, Applied Biosystems).

### Accession numbers of the gene sequences

The sequences determined in this work were deposited in GenBank under accession numbers KJ616748-KJ616751 for *rtxA*, KJ522612-KJ522619 for *gyrase A*, KJ522620-KJ522626 for *rstB*, KJ647311 and KJ652003-KJ652005 for *ctxAB* promoter region.

## Results

### Emergence of *rtxA*-null mutation in recent El Tor variant strains

**Figure 1:**
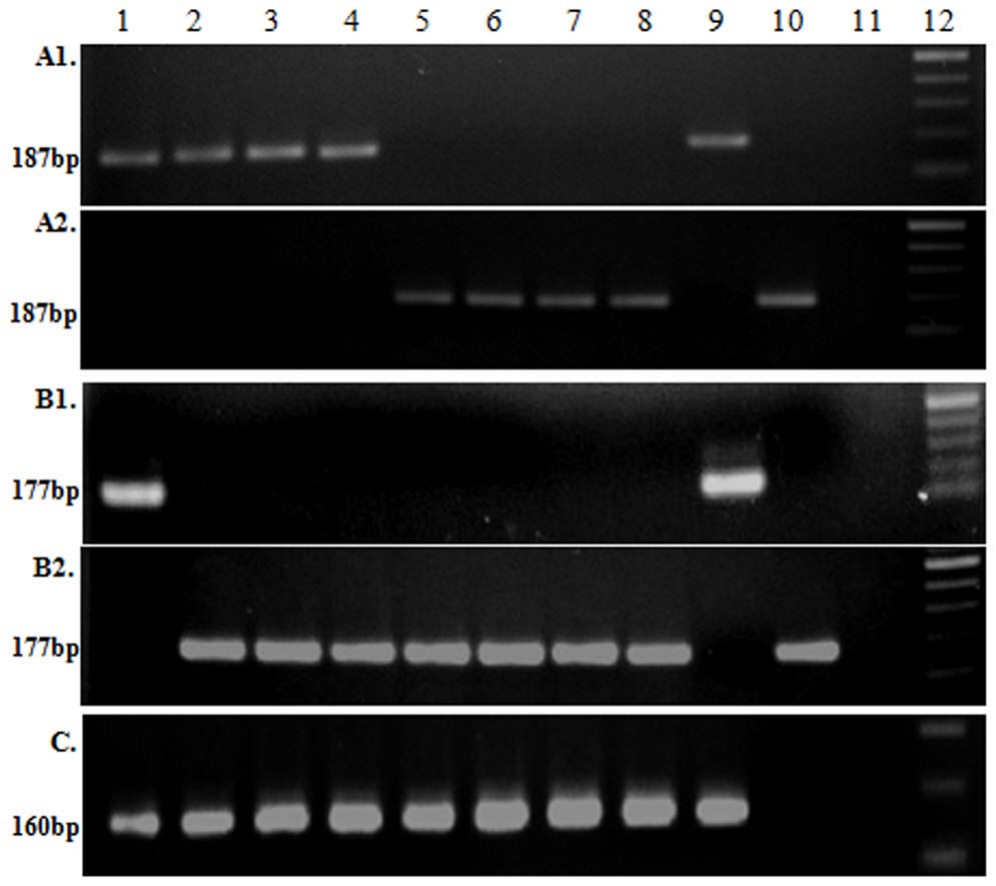
Development of PCR based assay for *rtxA*, *gyraseA* and *rstB* alleles in *V. cholerae* O1 Kolkata isolates. MAMA-PCR to detect the type of *rtxA* allele in representative *Vibrio cholerae* O1 strains of Kolkata using primers (rtxAF/rtxAR1) for El Tor (**A1**) and (rtxAF/rtxAR2) for Variant (**A2**). Lanes 1–8 represent RC60 (2001), SC46 (2003), J3752 (2004), K22638 (2005), J22384 (2004), L16899 (2006), IDH03044 (2010), IDH03672 (2011), respectively. To detect the type of *gyrase A* allele in representative *Vibrio cholerae* O1 strains of Kolkata using primers (gyrAF/gyrAR2) for El Tor (**B1**) and (gyrAF/gyrAR1) for Variant (**B2**). Lanes 1–8 represent V114 (1991), SC46 (2003), J3752 (2004), K22638 (2005), L16899 (2006), IDH03044 (2010), IDH03672 (2011), IDH01376 (2009), respectively. To detect the type of *rstB* allele in representative *Vibrio cholerae* O1 strains of Kolkata using primers rstBF1-rstBR2 (**C**). Lanes 1–8 represent RC60 (2001), SC46 (2003), J3752 (2004), K22638 (2005), L16899 (2006), IDH03044 (2010), IDH03672 (2011), IDH01376 (2009), respectively. In all cases N16961 (Lane 9) and El-1786 (Lane 10) were used as control for El Tor and Variant strain respectively. Lane 11 (DH5α) serves as negative control in all cases. The extreme right lane contains a 100- bp size ladder ((New England Biolabs Inc., Beverly, MA, USA).

**Figure 2:**
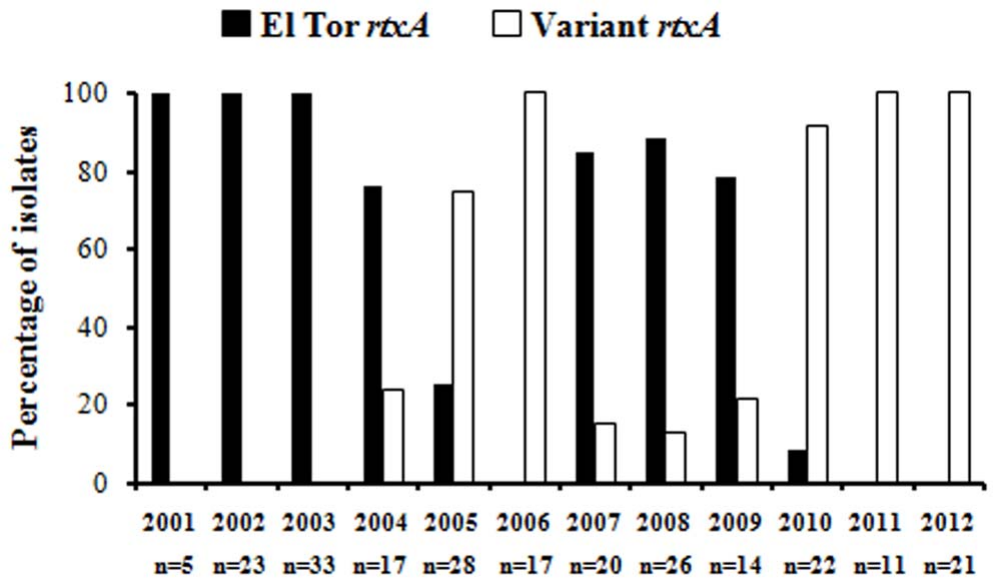
Retrospective analysis of *rtxA* allele in *V. cholerae* O1 Kolkata isolates. Occurrence of *rtxA* allele type in Kolkata *Vibrio cholerae*O1 strains from 2001 to 2012. A total of 237strains were tested during the study period and “n” denotes the number of strains tested in each year. *V. cholerae* O1 strains with variant *rtxA* was isolated in Kolkata for the first time in the year 2004.

The *rtxA* gene is the largest open reading frame (ORF) of the *V. cholerae* genome encoding a multifunctional auto processing RTX (MARTX) family toxin [24],[25]. Within the recently emerged Haitian outbreak strains, a mutation occurred in *rtxA* that introduces a premature stop codon that disabled the toxin function [15]. Using this sequence polymorphism, a simple PCR based assay was designed to understand the emergence of the *rtxA*-null mutation among the *V. cholerae* strains isolated in Kolkata. This newly developed PCR assay successfully differentiated the two different alleles of *rtxA* (Figure 1). PCR result was further confirmed by sequencing analysis using different set of primers (Table 1). We screened 237 *V. cholerae* O1 strains isolated from Kolkata covering different months of each year from 2001 to 2012 using this PCR assay. This analysis delineates the first appearance of an *rtxA*-null mutation (variant *rtxA*) in the Kolkata strains isolated during 2004. Although the variant *rtxA* totally displaced the El Tor *rtxA* in 2006, but the El Tor *rtxA* became dominant again during 2007. From 2011 onwards, the El Tor *rtxA* was totally replaced by the variant *rxtA* (Figure 2).

Subsequent to the emergence of an *rtxA* variant, another important mutation apparently occurred in strains with *rtxA*-null background, is the point mutation in CtxB (Asn^20^). We could not detect a single strain containing variant *ctxB* (*ctxB7*) in the El Tor *rtxA* background suggesting the emergence of variant *ctxB* (*ctxB7*) within the *rtxA*-null background.

The most important clinically relevant change in the *rtxA* toxin gene was one SNP that emerged within the currently existing El Tor variant strains. This SNP deactivated the function of the RTX toxin by incorporating a premature stop codon that had resulted a truncated protein by 12 amino acids, possibly affecting the C-terminal secretion signal.

### Lineage of *gyrA* of the Haitian isolates and the newer variant Kolkata isolates

**Figure 3:**
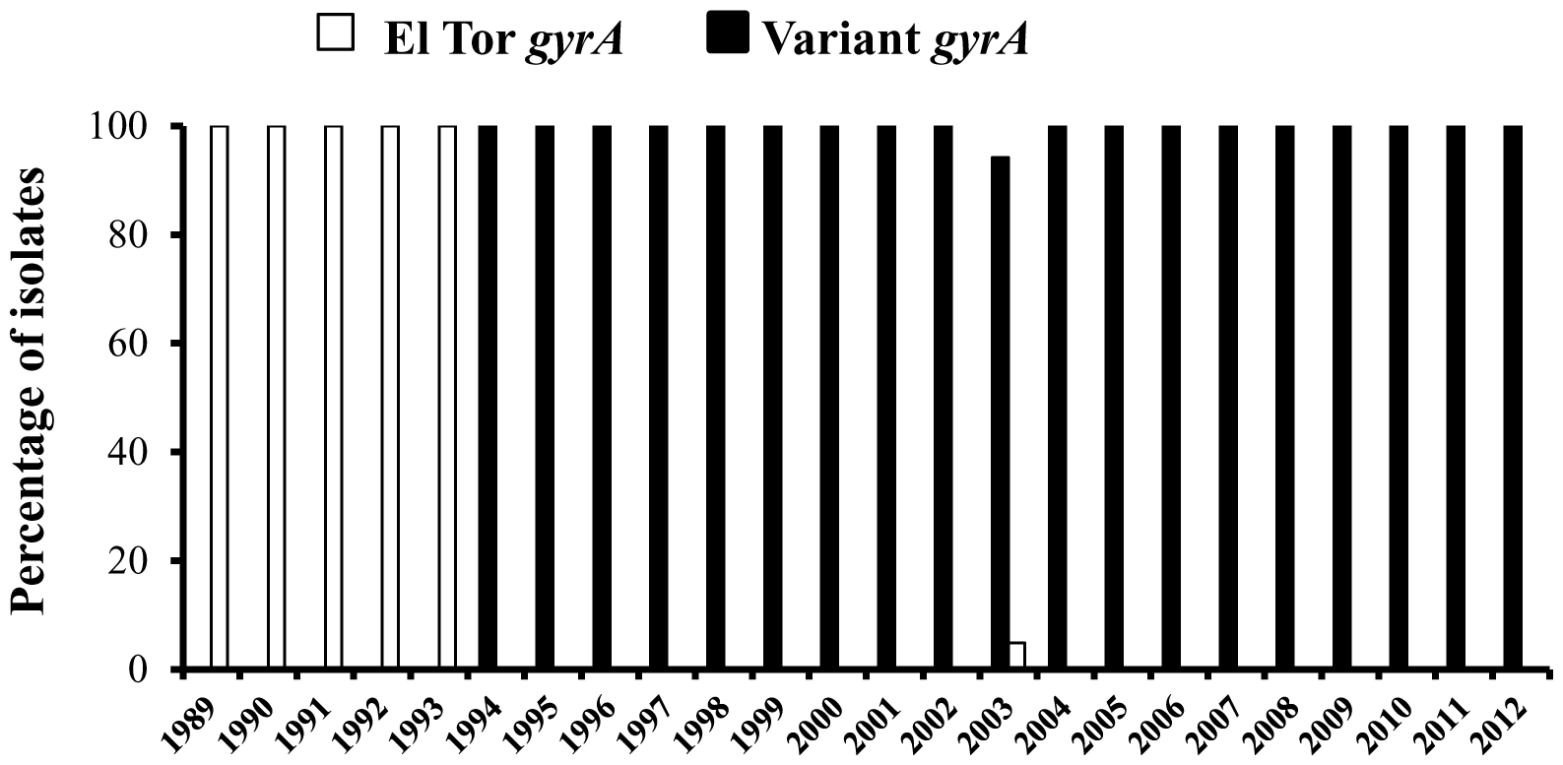
Isolation profile of *gyrA* allele in Kolkata. Occurrence of *gyrA* allele type in Kolkata *Vibrio cholerae* O1 strains from 1989 to 2012. A total of 287strains were tested during the study period. *V. cholerae* O1 strains with variant *gyrA* was isolated in Kolkata for the first time in the year 1994.

The targets of the quinolones are the type II topoisomerases DNA gyrase, a heterotetramer composed of two A and two B subunits, encoded by the gyrase A and gyrase B gene respectively [26]. Analysis of the *gyrA* gene of the Haitian strains documented a Ser83→Ile substitution which is associated with quinolones resistance in clinical *V. cholerae* [16]. Focusing on the sequence polymorphism a PCR assay was done using allele specific primers (Table 1). After standardization of the PCR (Figure 1), sequencing of the representative strains isolated from Kolkata was performed to reconfirm the PCR assay. A retrospective analysis of the 287 *V. cholerae* strains isolated between 1989 and 2012 using this PCR indicated that a variant *gyrA* allele was first introduced in Kolkata during 1994. Interestingly, soon after its appearance, this *gyrA* variant totally displaced the El Tor *gyrA* allele (Figure 3). These results not only showed the prevalence of the *gyrA* lineage among the Haitian isolates but also reveal that this allele exists in Kolkata from 1994 onwards.

### Analysis of the *rstB* gene and the ToxR binding repeats of the Kolkata isolates

A GTA deletion at nucleotide positions 77–79 in the *rstB* of the Haitian outbreak strain has been documented [16]. In order to screen these deletions if any in Kolkata strains, primers were designed for a PCR assay. None of the newer variant Kolkata isolates had any deletions in the *rstB* gene (Figure 1). Sequencing study reassured the PCR based result. This genotypic assessment underscored that the genomic feature of *rstB* gene of Kolkata isolates does not resemble the *rstB* gene of Haitian *V. cholerae* strains.

**Figure 4:**
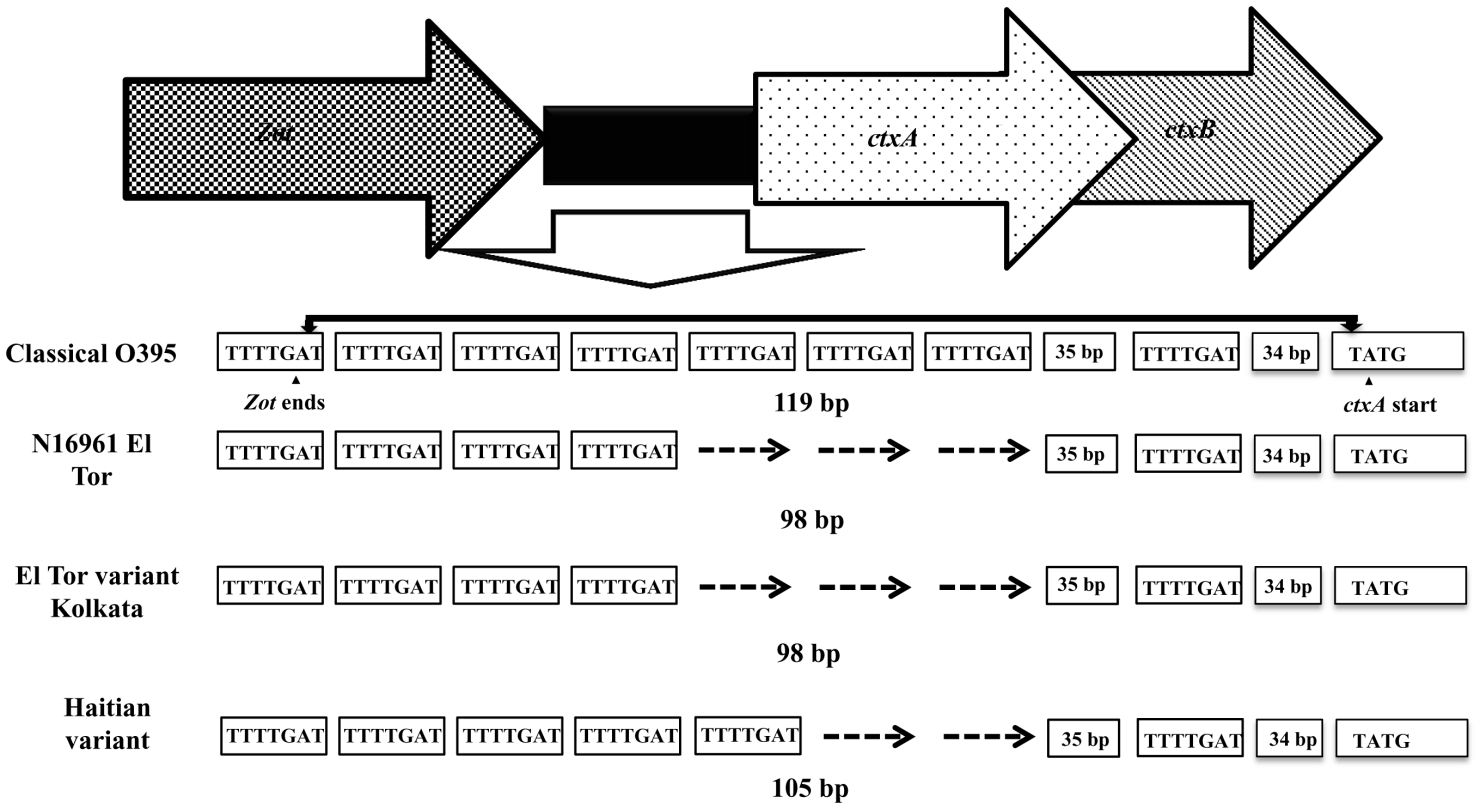
Schematic representation of the promoter region the *ctxAB* operon of *Vibrio cholerae* O1 El Tor variant strains isolated from Kolkata and Haiti and its comparison with classical and El Tor strains. The Kolkata strains contained four heptad repeats (TTTTGAT) in their CT promoter region whereas Haitian strain carried 5 heptad repeats. The dashed arrow (- - - - →) denotes the lack of repeat regions.

Variation in the number of repeats (TTTTGAT) in the ToxR binding region between *zot* and *ctxA* has been reported earlier. Whole genome sequence analysis of the Haitian outbreak strain El–1786 showed five copies of the heptads repeat in the ToxR binding region [14]. Our sequence based results confirmed presence of four heptad repeats in Kolkata strains (Figure 4).

## Discussion

Whole genome sequence analysis of the Haitian *V. cholerae* strain contained a mutation at the 89^th^ amino acid position of the whole TcpA subunit and at the 20^th^ amino acid of CTB [13],[14],[27]. Though these mutations were also reported from different parts of the world but they were highlighted after the catastrophic cholera outbreak in Haiti. All the *V. cholerae* strains in Kolkata had classical *ctxB* with El Tor *tcpA* up to September 2003. Our previous study indicated that the variant *tcpA* and *ctxB* first appeared in Kolkata during October 2003 and April 2006, respectively [4],[23]. It means that all the *V. cholerae* strains in Kolkata had classical *ctxB* with El Tor *tcpA* up to September 2003. Then the combination changed to classical *ctxB* with variant *tcpA* from October 2003 onwards. Finally, certain proportion of the *V. cholerae* strains in Kolkata acquired the combination of variant *ctxB* (*ctxB7*) with variant *tcpA* (*tcpA* CIRS) from April 2006 onwards. To understand the prevailing genomic features of the Kolkata isolates and the relatedness with Haitian *V. cholerae* strains, SNP analysis was undertaken in this study. Our retrospective analysis showed that the Kolkata variant shared some of the genetic traits with the Haitian cholera outbreak strains.

Presence of variant *rtxA* (*rtxA*-null mutation) in the altered El Tor Bangladesh isolates in the early phase of 1999, India in 2004 and Haiti in 2010 was reported [11],[15],[28]. Utilizing the existing sequence polymorphisms, a simple PCR and sequence based retrospective analysis was conducted to identify the emergence and dissemination of the *rtxA*-null mutation in the Kolkata isolates. Appearance of *rtxA*-null mutation in the Kolkata isolates was first detected in 2004 with subsequent appearance of the*ctxB*7 allele in 2006 [4]. This null mutant may be the genetic background for the subsequent emergence of the *ctxB7* allele as not a single strain containing the combination of El Tor *rtxA* and *ctxB7* was detected in our study from Kolkata. Interestingly, the classical strains are also known to have a deletion that removes>7 kb of the *rtx* locus, deactivating the RTX [29]. The epidemiological advantage of deletion of RTX in recent *V. cholerae* O1 strains are not yet known. The toxin is very large and may be harmful to growth due to energy expenditure reducing rapid growth necessary for increased dissemination.

It was reported that the Haitian strains have a Ser83→Ile substitution in the *gyrA*, which is associated with quinolone resistance in clinical *V. cholerae* strains [16]. This mutation was also reported from India, Nigeria and Cameroon [30]–[34]. Such genetic events were recorded in Kolkata strains since 1994. An additional feature of the Haitian strains was the GTA deletion in the *rstB* [16]. Our PCR and sequence results showed that this deletion, a unique feature for a classical biotype of *V. cholerae* [35], was not detected in any of the Kolkata isolates. Sequence based analysis has demonstrated the presence of four copies ToxR binding repeats (TTTTGAT) in Kolkata isolates in comparison with the five copies of repeats in Haitian isolates. Finally, our retrospective analysis conveyed that few genetic traits of *V. cholerae* O1 Haitian isolates are absent in contemporary strains from Kolkata, India. Genomic analysis of *V. cholerae* isolates obtained from the early phase of Haitian cholera epidemic has provided evidence that the Haitian isolates derived from a strain similar to *V. cholerae* isolated in South Asia. [7],[11],[36],[37]. Using the limited information on comparison of genomes, Chin *et al*., (2011) concluded that the Haitian cholera outbreak was most likely due to the introduction of a strain from a remote cholera endemic region [10]. An Independent Panel of Experts from the United Nations prepared a report regarding the cholera outbreak in Haiti during 2010 and opined that this cholera outbreak was due to the contamination of upstream water in the Artibonite River with a pathogenic strain that had genetic resemblance with the *V. cholerae* strain from South Asia [38]. The origin of the Haitian outbreak further became complex with whole genome sequence analysis that indicated Nepal as the likely origin of the Haitian outbreak. [11]. Katz *et al* (2013) with the help of genomic comparisons of 108 *V. cholerae* genomes from Thailand, Bangladesh, Nepal, Cameroon, India, Pakistan, and Benin narrated that Haitian isolates were not only nearly identical to the isolates from Nepal but also the Nepal-Haiti isolates were clearly distinct from isolates circulating elsewhere in the world [39]. *V. cholerae* strain responsible for the cholera epidemic in Haiti had similar phenotypic and genetic properties of the seventh pandemic El Tor O1 strains [7],[28],[37]. In depth studies that used whole genome phylogeny and core genome SNPs have shown the genetic relations of Haitian outbreak strain with strains originated from India and Cameroon [9].

Considering several reports on Haitian cholera epidemic, our study reveals that Haitian variant strain may be modulated as a result of the sequential genetic events in the Indian subcontinent strain. These genomic events came into focus after Haitian outbreak and these changes reigned erstwhile before the Haitian disaster. The unique genetic attributes of *rstB* and the *ctxAB* promoter repeat not only signify a cryptic change in the genome of *V. cholerae* O1 but also highlighted particular genetic traits of Kolkata isolates that are different from the Haitian outbreak strains. Finally, our findings convey the message about the similarity for several of the newer genetic traits of the Haitian isolates. The time of appearance of these genetic traits and the dissimilarities in genomic content with respect to *rstB* and *ctxAB* promoter repeat though knocking the particular genetic traits of Kolkata isolates but also enlighten the belief that Haitian variant strain may be result of the sequential event in the evolution of Indian subcontinent strain with some cryptic modification in the genome. This hypothesis requires several strain characteristics, epidemiological and experimental validations.
